# Endovascular Repair of Aortobronchial Fistula due to Saccular Aneurysm of Thoracic Aorta

**DOI:** 10.1155/2017/3158693

**Published:** 2017-11-27

**Authors:** Konstantinos Tigkiropoulos, Kyriakos Stavridis, Ioannis Lazaridis, Nikolaos Saratzis

**Affiliations:** Vascular Unit, 1st University Department of Surgery, Aristotle University of Thessaloniki, Papageorgiou General Hospital, Thessaloniki, Greece

## Abstract

Aortobronchial fistula (ABF) is a rare condition which can be lethal if left untreated. Open surgical treatment carries high morbidity and mortality. Recent advances in endovascular technology have made thoracic endovascular aortic repair (TEVAR) the treatment of choice. We present a successful endovascular repair of aortobronchial fistula due to a saccular aneurysm of descending thoracic aorta.

## 1. Introduction

Aortobronchial fistulas are communications between the thoracic aorta and the adjacent pulmonary parenchyma or tracheobronchial tree. The causes include aortic aneurysms, pseudoaneurysms, traumatic thoracic aorta injuries, penetrating aortic ulcers, and previous thoracic aortic surgery [1–3]. Intermittent or massive haemoptysis is the main symptom, but it is nonspecific and high index of suspicion is necessary to diagnose ABF. Conventional open surgical treatment was the treatment of choice for many years and is associated with increased morbidity and mortality with the latter ranging from 15 to 41% [1, 4]. Recent advances in endovascular technology have made TEVAR the treatment of choice, with success rate of 90% and in-hospital mortality of 3%, for multiple aortic pathologies including aneurysms, dissections, traumatic aortic injuries, and aortobronchial as well as aortoesophageal fistulae [5]. We present the successful repair of an ABF in a patient using endoluminal self-expandable stent grafts.

## 2. Case Report

**Figure 1:**
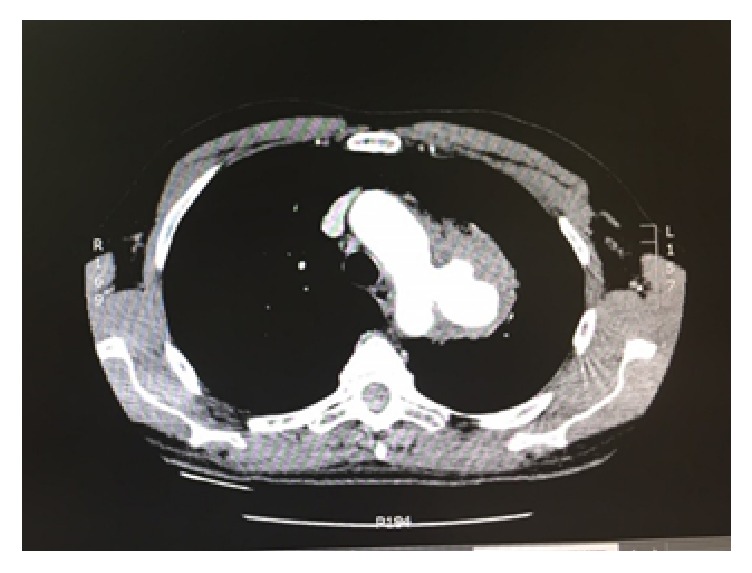
CT with intravenous contrast showed a 5,5 cm saccular aneurysm of descending thoracic aorta.

**Figure 2:**
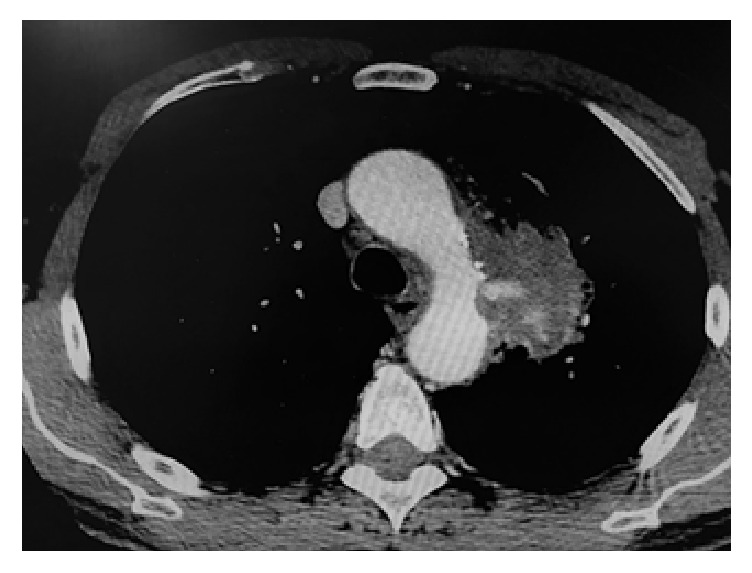
CT depicted an aortobronchial fistula with intrapulmonary hematoma.

A 64-year-old male presented to the emergency with a massive heamoptysis. He had a history of interscapular pain and intermittent haemoptysis the last five days. Laboratory values were normal apart from hematocrit (Hct = 26%). An urgent computer tomography (CT) with intravenous contrast was performed which showed a 5,5 cm saccular aneurysm of the descending thoracic aorta arising less than 2 cm from the left subclavian artery and intrapulmonary hematoma (Figures 1 and 2). Preoperative bronchoscopy was avoided since it could provoke an intrapulmonary bleeding which could be fatal. Immediate consult for vascular surgery was called.

**Figure 3:**
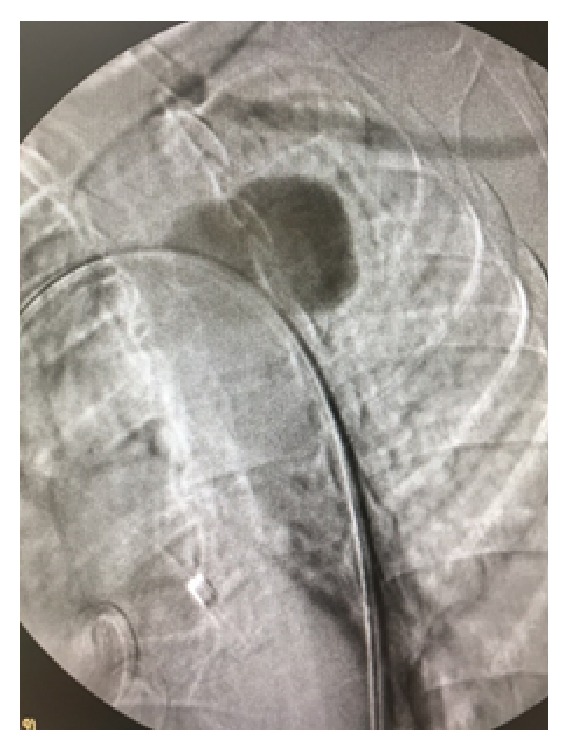
DSA revealed the presence of pseudoaneurysm just distal to the left subclavian artery.

**Figure 4:**
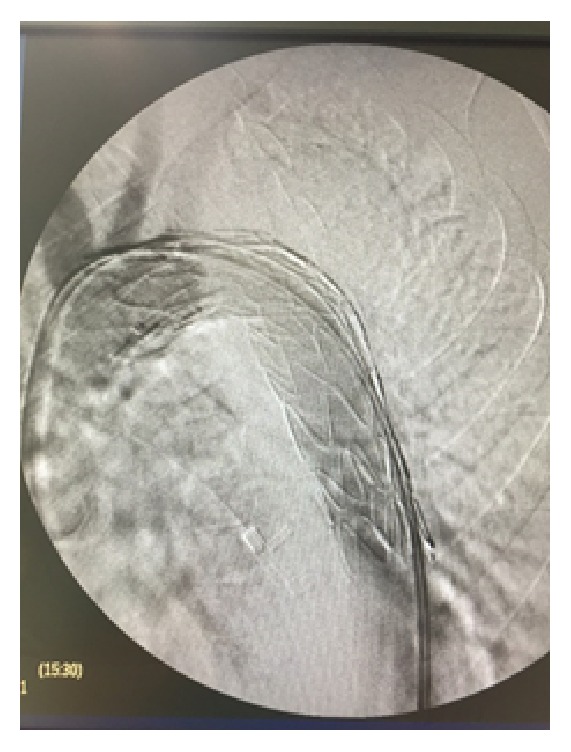
Deployment of Ankura endograft with exclusion of the saccular aneurysm, coverage of the left subclavian artery, and absence of endoleak.

The procedure was carried out in a fully equipped operation theatre under spinal anaesthesia. Surgical access was gained through common femoral arteries in standard surgical fashion. The patient was systematically heparinized (75 units/kg). A Terumo guidewire (Terumo Corporation, Tokyo, Japan) was inserted through the right common femoral artery to the ascending aorta which was exchanged with a 0.035 Backup Meier (Boston Scientific, USA) stiff wire. An 8F-60 cm arrow (Arrow International, Inc., Reading, PA, USA) was advanced through the left common femoral artery to the descending thoracic aorta. An aortography was performed which revealed the saccular aneurysm just distal to the left subclavian artery (Figure 3). An Ankura (Lifetech Scientific; Maastricht, Netherland) endovascular stent graft with 40 mm diameter and 16 cm length was deployed under fluoroscopic control. Completion arteriogram was performed with exclusion of the saccular aneurysm, coverage of the left subclavian artery, and absence of endoleak (Figure 4).

**Figure 5:**
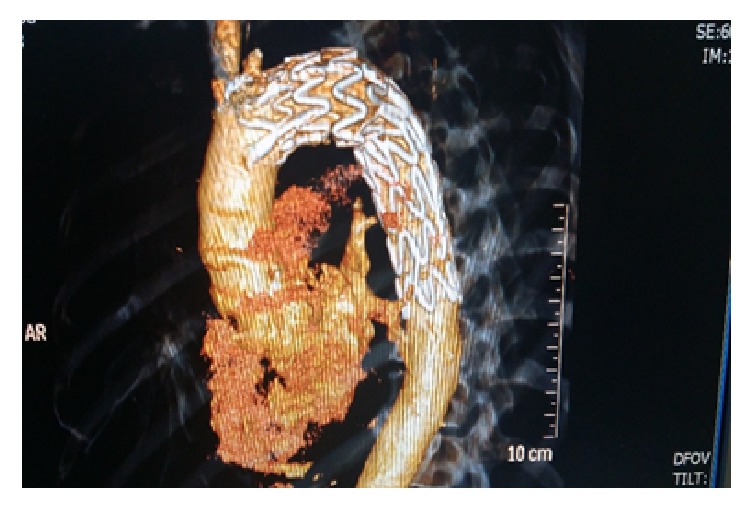
Three-dimensional CT angiography of thoracic aorta with proper placement of endograft and occlusion of left subclavian artery.

The patient had an excellent recovery with no signs of arm ischemia postoperatively. He was discharged after 7 days under a single antiplatelet therapy (clopidogrel) and a broad spectrum antibiotic therapy (moxifloxacin) for at least 3 months. A follow-up CT angiography revealed accurate placement of endograft with no endoleak and thrombosis of the saccular aneurysm (Figure 5). A PET scan with 8 fluorodeoxyglucose was performed 1 month postoperatively which was negative for endograft infection.

## 3. Literature Review

A systematic review by Riesenman et al. at 2009 [6] reported published case reports and case series of endovascular repair of aortobronchial fistulas. Sixty-five patients were treated with stent graft deployment. Most of the patients (55%) had previously undergone thoracic aortic surgery, and of these 36 patients 14 (39%) had undergone the initial aortic operation for aneurysms or pseudoaneurysm of the descending thoracic aorta and 13 (36%) for congenital aortic coarctation. A commercially manufactured thoracic endograft was deployed in 75% of patients and reintervention was performed in 8 patients, 7 of these in less than 30 days from the procedure.

Canaud et al. provided a systematic review of outcomes of thoracic endovascular repair for aortobronchial fistula [7]. One hundred thirty-four patients were identified. TEVAR was performed within 24 hours in 84.8% of the patients. Overall 30-day mortality was 5.9%, endovascular leak was the main complication, and recurrence of ABF was observed in 14 patients (11.1%). Midterm outcomes (after 30 days) included all-cause and aortic related mortality which were 21.4% and 14.3%, respectively.

## 4. Discussion

Aortobronchial fistula (ABF) is a potentially serious condition which can be lethal if it is not diagnosed or left untreated [2, 4]. Most of the times it is difficult to diagnose but a high index of suspicion is essential in patients with a known history or previously treated thoracic aortic pathology. Various imaging techniques can assist in the diagnosis of ABF. Computed tomography angiography (CTA) is the imaging technique of choice due to its accuracy and speed [8]. The cardinal symptom is haemoptysis, intermittent or massive, which may be fatal [9, 10].

Once diagnosis of ABF has been established prompt intervention is vital. Open surgical repair was the mainstay of treatment for decades; however it is associated with high morbidity and mortality due to the need for emergent operation in patients with multiple comorbidities, reoperative thoracic aortic exposure, possible cardiopulmonary bypass, and aortic cross clamping [2, 11]. Picichè et al. provided a review of specifically postoperative aortobronchopulmonary fistulas. Seventy-six patients were identified with a total of 79 fistulas. Open surgical procedures were used in 50 fistulas with a mortality rate of 16%, most of them dying intraoperatively. In the group of patients (*n*: 15) who were treated endovascularly there was not any death intraoperatively and author concluded that endovascular treatment was the proper therapy [12].

TEVAR has become the treatment of choice for multiple aortic pathologies including ABFs with low rates of mortality, 3%, shorter hospitalization in the intensive care unit, and faster rehabilitation [5]. However, due to its rarity, experience with endovascular repair is limited and outcomes have been reported to small case series or isolated case reports [13–22]. Further studies are needed to determine its long term efficacy and safety.
